# A choice motif

**DOI:** 10.7554/eLife.19351

**Published:** 2016-08-18

**Authors:** Sabine L Renninger, Michael B Orger

**Affiliations:** Champalimaud Neuroscience Programme, Champalimaud Centre for the Unknown, Lisbon, Portugal; Champalimaud Neuroscience Programme, Champalimaud Centre for the Unknown, Lisbon, Portugalmichael.orger@neuro.fchampalimaud.org

**Keywords:** motor control, escape, inhibitory interneurons, Zebrafish

## Abstract

A simple neural circuit motif in the zebrafish brain enables robust and reliable behavioral choices.

**Related research article** Koyama M, Minale F, Shum J, Nishimura N, Schaffer CB, Fetcho JR. 2016. A circuit motif in the zebrafish hindbrain for a two alternative behavioral choice to turn left or right. *eLife* **5**:e16808. doi: 10.7554/eLife.16808

**Image** Inhibitory neurons (red) connect to Mauthner neurons (blue) in a circuit that allows zebrafish to turn rapidly away from sounds

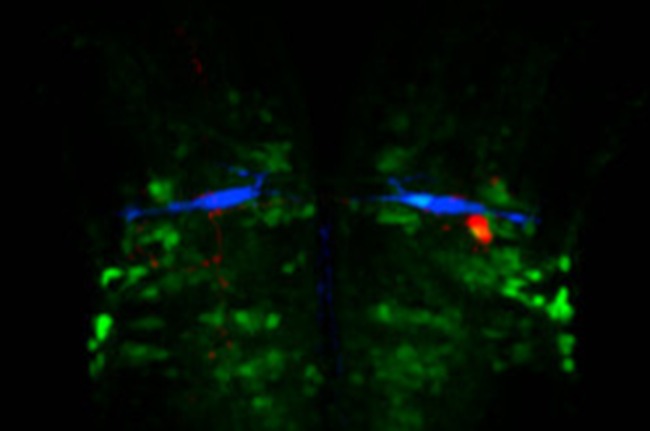

Understanding how vertebrates execute even simple behaviors is a daunting task, given that their brains consist of networks of hundreds of thousands to billions of neurons. Recent technical advances in methods to record and manipulate neural activity have made it easier to study these networks. However, experimental access is not sufficient to provide an understanding of complex systems (Lazebnik, 2002).

Can neurobiologists, like electrical engineers, gain insight by becoming familiar with a set of simple components from which more complex systems are constructed? One approach is to identify network motifs – patterns of connectivity among small groups of neurons that, due to their useful or robust dynamic properties, appear frequently in different neural systems (Alon, 2007). However, there are relatively few such motifs whose properties are well understood. Now, in eLife, Joseph Fetcho and colleagues of Cornell University and the Janelia Research Campus – including Minoru Koyama as first author – have characterized a circuit motif, in which inhibitory neurons inhibit each other, that allows zebrafish larvae to make rapid, reliable and accurate choices based on sensory inputs (Figure 1; Koyama et al., 2016).

**Figure 1.**
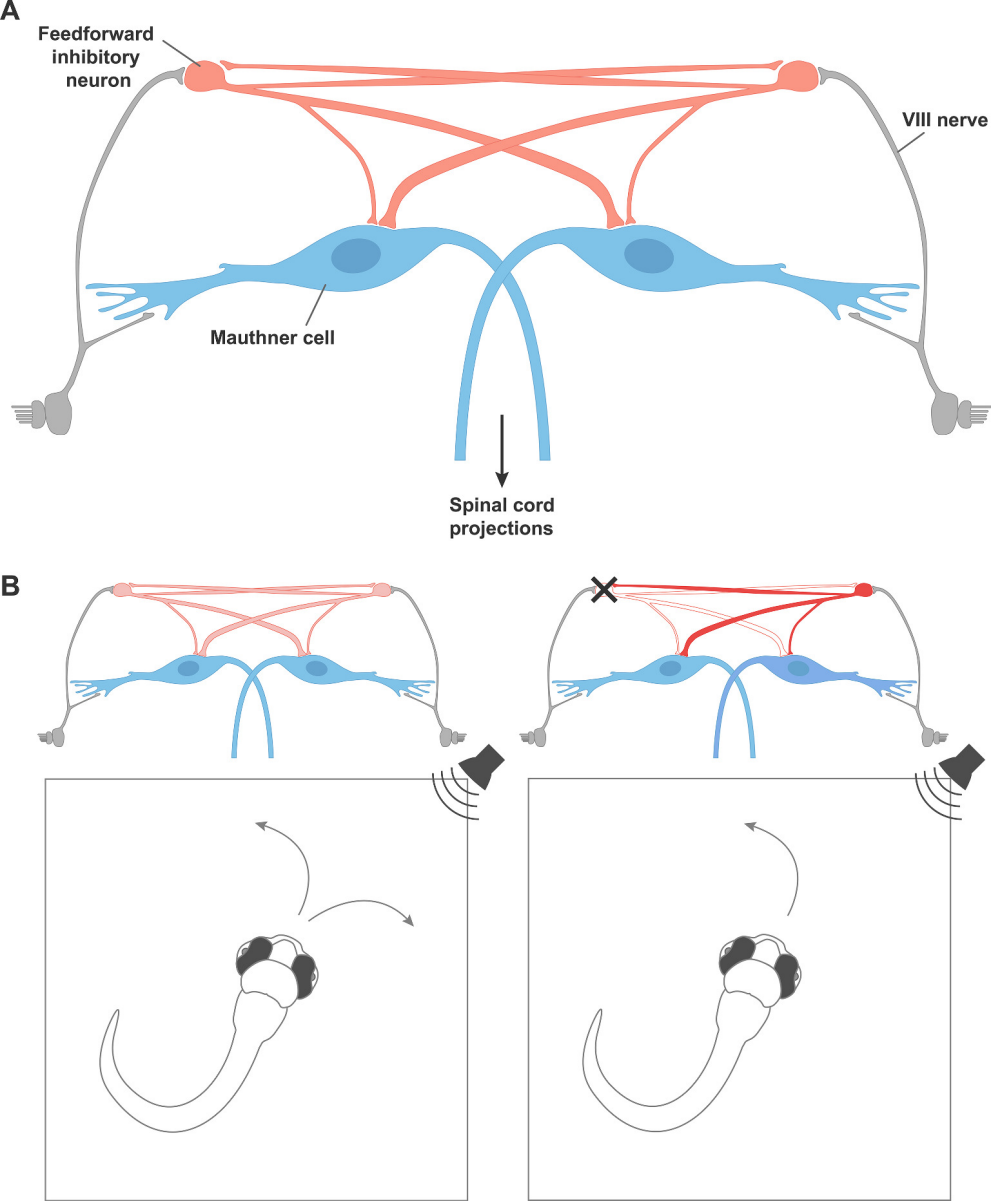
A circuit motif that underlies behavioral choices in zebrafish. (**A**) A neural circuit motif in the zebrafish brain that controls the choice to escape left or right, as described by Koyama et al. Auditory signals from cranial nerve VIII (grey) excite the giant Mauthner neurons (blue), which transmit this information down the opposite side of the spinal cord via their axons in order to drive a rapid turn away from the stimulus. Cranial nerve VIII also excites feedforward inhibitory neurons (red), which connect with the Mauthner neurons on both sides, but connect more strongly to the Mauthner neuron on the opposite side of the brain. The feedforward inhibitory populations on each side of the brain also inhibit each other. (**B**) Left: In response to a non-localized auditory stimulus, fish turn to the left or right with equal probability. Right: destroying the feedforward inhibitory neurons on the left disinhibits the feedforward neurons on the right side of the brain. This leads to stronger inhibition of the Mauthner neuron on the left. The fish is therefore more likely to turn to the left, thanks to the firing of the right Mauthner neuron.

Fish can escape threatening stimuli with extraordinary speed, turning away within milliseconds of first hearing or otherwise sensing a threat. The turn must be both fast and in the correct direction. This behavior is controlled by a specialized circuit of neurons that includes a pair of giant cells called the Mauthner neurons. These cells are found in the hindbrain of fish and amphibians, with one neuron on the left side of the brain and the other on the right (Korn and Faber, 2005), and similar giant neuron systems have been implicated in escape behavior in animals ranging from invertebrates to mammals (Lingenhöhl and Friauf, 1994; Wiersma, 1947). The axon of each Mauthner neuron crosses the midline of the brain so that it passes down the spinal cord on the opposite side of the body (i.e. the axon of the Mauthner neuron on the left side of the brain passes down the right side of the spinal cord, and vice versa).

A single spike of electrical activity in one Mauthner neuron is sufficient to trigger a powerful muscle contraction and start an escape swim. When responding to loud sounds, the brain needs to gauge small differences between the strength of the input signal received from each ear to select the appropriate way to turn. Strong feedback mechanisms ensure that usually only one Mauthner neuron fires during an escape (although see Satou et al., 2009).

To define the neural circuit that determines which Mauthner cell fires, Koyama et al. focused first on a particular population of genetically labeled inhibitory neurons. These neurons receive inputs about sounds from auditory fibers and connect with other neurons on both sides of the brain.

Koyama et al. made whole-cell patch clamp recordings from individual inhibitory neurons and both Mauthner cells simultaneously. This revealed that many of the neurons made direct connections to both Mauthner cells, although the connection to the Mauthner cell on the opposite side of the brain was usually stronger. This asymmetry causes greater suppression of the Mauthner cell on the side of the fish that receives weaker input from the stimulus, and could favor turns in the correct direction. For example, if a sound is heard on the left side of the fish, the Mauthner cell on the right is suppressed more strongly. Thus the Mauthner cell on the left is more likely to fire, causing the muscles to contract on the right side of the body, and leading the fish to turn to the right.

To make these arguments quantitative, Koyama et al. built a computer model of the circuit. Incorporating the observed asymmetric inhibition into the model ensures that the model fish turn in the correct direction. However, in response to intense stimuli that were audible on both the left and the right, neither Mauthner neuron fired at all, due to the strong inhibition on both sides. How do real fish avoid this unfortunate outcome?

Koyama et al. hypothesized that if the populations of inhibitory neurons on either side of the brain mutually inhibited each other, this would reduce the total inhibition. Modifying the model to incorporate mutual inhibition confirmed this intuition: strong stimuli could now produce appropriately directed turns. Moreover, patch clamp recordings from pairs of inhibitory neurons on both sides of the brain verified the existence of these crossed connections in living fish. Circuits that feature such reciprocal inhibition of inhibition have been shown theoretically to be optimal for rapidly and reliably categorizing inputs (Mysore and Knudsen, 2012), which is the precise goal of the Mauthner network.

A good model should be able to predict how a system will respond to a new set of conditions, but this is something that is rarely possible in a behaving animal. Fortunately, in the small transparent brain of the zebrafish larva, it is possible to selectively destroy single neurons using lasers, and observe the effects on behavior. The model of Koyama et al. makes two predictions of what should happen if the inhibitory neurons on one side are selectively removed. First, the response should occur even faster than before, since the activity of the inhibitory neurons delays the firing of the Mauthner neuron. Second, the fish should be more likely to turn towards the side on which the inhibitory neurons have been destroyed. Experiments performed on zebrafish larvae confirmed these predictions, providing convincing proof that Koyama et al.’s model has captured the essential features of connectivity and dynamics in this small circuit.

The ability to weigh sensory evidence and rapidly choose between two outcomes is fundamental to many behaviors, and there is good reason to expect that the circuit features observed by Koyama et al. will have relevance beyond the Mauthner cell circuit. Reciprocal inhibition of inhibitory neurons has been observed in other systems, including circuits in the avian midbrain that are involved in competitive stimulus selection (Goddard et al., 2014). In other biological networks, connectivity motifs that have robust and useful dynamical properties are found with greater abundance than less stable ones (Prill et al., 2005). Moreover, the relevant inhibitory neurons were identified and targeted based on previous work from the same group, which showed that transcription factor stripes in the hindbrain define different categories of neurons with distinct projection patterns and neurotransmitter types (Kinkhabwala et al., 2011; Koyama et al., 2011). These form a common set of building blocks for the circuits that control different behaviors.

This work, together with other recent studies (LaCoste et al., 2015; Neki et al., 2014), shows that the Mauthner circuit, after many decades of investigation, continues to provide fertile ground for new insights into the neural circuit mechanisms that control behavior.
